# 

**Published:** 1901-05

**Authors:** 


INTERNATIONAL DENTAL PUBLICATION CO.,

624 Chestnut Street, Philadelphia, Pa.

I desire to subscribe to the

### INTERNATIONAL DENTAL JOURNAL, commencing with the..........................................19 issue, and until otherwise instructed, for which I agree to pay the subscription price of $2.50 per year.

.................................................. Name.

.................................................. P.O.

.................................................. State.

